# The acidic tumor microenvironment in hepatocellular carcinoma: a barrier to adoptive cellular immunotherapy

**DOI:** 10.3389/fimmu.2026.1959235

**Published:** 2026-09-22

**Authors:** Jingnan Xiong, Guozirui Mou, Taduoji Ze, Xia Li, Hongyun Zhao

**Affiliations:** 1 Department of Gastroenterology, The Second Affiliated Hospital of Chongqing Medical University, Chongqing, China; 2 The First Department of Internal Medicine, the People’s Hospital of Qamdo, Tibet Autonomous Region, China

**Keywords:** acidic tumor microenvironment, adoptive cellular immunotherapy, CAR-T cells, hepatocellular carcinoma, immune evasion, immunometabolism, lactylation, tumor-associated macrophages

## Abstract

The metabolic interface between tumor and immune cells is an emerging determinant of immunotherapy resistance, yet its therapeutic exploitation in hepatocellular carcinoma (HCC) remains limited. Adoptive cellular immunotherapies, including NK cell infusion, tumor-infiltrating lymphocytes (TILs), and chimeric antigen receptor T (CAR-T) cells, have transformed hematologic oncology but repeatedly fail to achieve durable responses in HCC. We argue that an underappreciated driver of this resistance is the acidic tumor microenvironment (TME): hypoxia-driven glycolytic reprogramming acidifies the HCC niche through lactate and proton accumulation, lowering extracellular pH (pHe) to approximately 6.5–6.8 (measured 6.66 ± 0.19 in human HCC by CEST MRI), rewiring macrophages, neutrophils, dendritic cells, NK cells, myeloid-derived suppressor cells, and T lymphocytes toward immunosuppressive or exhausted states. Although lactate and tumor acidity have each been reviewed extensively, the specific implications of acidosis for the design and delivery of cell-based therapies remain scattered and unsynthesized, a gap of growing urgency as the first pH-targeting and acidity-armored cellular products enter clinical testing, as summarized in this review. This mini review delineates how the acidic TME is established in HCC, summarizes cell-type-specific mechanisms of acidosis-driven immune dysfunction, and critically evaluates unresolved issues: the incomplete dissociation of acidity- versus lactate-mediated effects, the metabolic double-edged sword of systemic glycolytic blockade, and the gap between preclinical pH modulation and clinical translation. We conclude that ex vivo metabolic preconditioning and acidity-responsive cell engineering, rather than systemic metabolic intervention, offer the most tractable path toward durable cellular immunotherapy in HCC.

## 1 Introduction

Hepatocellular carcinoma (HCC) remains a leading global malignancy, ranking as the second cause of cancer-related death in China (1, 2). Curative options are limited: surgical resection is applicable to only a minority of patients at diagnosis, and recurrence rates reach approximately 70% within 5 years even in this cohort (3). Although immune checkpoint inhibitors (ICIs) have improved the management of advanced HCC, response rates remain suboptimal compared with other solid tumors, largely because of a profoundly immunosuppressive tumor microenvironment (TME) (4). Adoptive cellular immunotherapy (ACI)—encompassing NK cell infusion, tumor-infiltrating lymphocyte (TIL) therapy, and chimeric antigen receptor T (CAR-T) cell therapy—has emerged as a complementary strategy, but its clinical durability in HCC has so far been modest (5).

The physicochemical barriers of the HCC TME, particularly extracellular acidification, have been reviewed elsewhere with a focus on tumor-intrinsic glycolysis, lactate signaling, and ICI resistance (6–8). What remains fragmented is a cell therapy-centered perspective: how the acidic niche quantitatively and mechanistically constrains each cellular platform, and which engineering or preconditioning strategies can confer acidic resilience. This mini review addresses that gap. We first outline how the acidic TME is established, then summarize its cell-type-specific effects on innate and adaptive immunity, and finally discuss adoptive cellular therapies through the lens of acidity—concluding with a critical appraisal of unresolved mechanistic and translational questions.

Terminological clarification. Throughout this review, we distinguish four concepts that are often conflated: (i) lactate accumulation, i.e., elevated lactate acting as a metabolite and signaling molecule; (ii) extracellular acidosis, i.e., reduced extracellular pH (pHe) from proton loading; (iii) intracellular acidification, i.e., reduced cytosolic pH (pHi); and (iv) redox imbalance, specified by the redox couple involved. These are not interchangeable: plasma membrane pH-regulatory systems—including NHE1, monocarboxylate transporters, and bicarbonate-dependent transporters—hold pHi within a narrow range even as pHe falls, so that tumor cells typically display a reversed pH gradient (pHi ≥ 7.2 versus pHe 6.5–6.9) (9, 10). Accordingly, statements below specify, wherever the underlying evidence permits, whether the causal driver is extracellular protons, lactate itself, or a defined redox perturbation.

## 2 Formation of the acidic TME in HCC

The HCC TME comprises malignant hepatocytes, heterogeneous immune infiltrates (T/B lymphocytes, TAMs), aberrant vasculature, activated stromal cells, and remodeled extracellular matrix (11, 12). HCC cells constitutively display aerobic glycolysis—the Warburg effect, first described by Otto Warburg a century ago—maintaining high glucose uptake and lactate production even when oxygen is plentiful (13). Superimposed on this constitutive program, as tumors outgrow their vascular supply and oxygen diffusion becomes limiting, hypoxia-inducible factor-1α (HIF-1α) is stabilized and drives a distinct hypoxia-induced glycolytic adaptation, upregulating glycolytic enzymes and transporters—including HK2, PFK1, PKM2, and GLUT1—to amplify glycolytic flux and lactate output (14, 15). Constitutive aerobic glycolysis and HIF-1α-dependent hypoxic adaptation frequently coexist in HCC but are biochemically distinct and should not be conflated (14). The resulting accumulation of lactate and protons lowers pHe to approximately 6.5–6.8: direct CEST MRI measurement in patients with liver cancer yielded a mean intratumoral pHe of 6.66 ± 0.19 (n = 15), versus 7.34 ± 0.09 in benign hepatic hemangioma (16), whereas values approaching 6.0—reported in extreme acidic regions of some solid tumors and experimental models—should not be regarded as representative of HCC. To survive this chronic acid load, HCC cells deploy an integrated pH-regulatory network: carbonic anhydrases CAIX/CAXII catalyze the reversible hydration of CO2 to facilitate proton extrusion and bicarbonate import; lactate dehydrogenase A (LDHA) regenerates NAD+ to sustain glycolytic turnover; monocarboxylate transporter 4 (MCT4)—the low-affinity, high-capacity isoform of highly glycolytic cells—mediates coupled lactate/H+ efflux from HCC cells, whereas the higher-affinity MCT1 predominates in lactate-importing cells, with flux direction set by the transmembrane lactate/H+ gradient (17, 18); and acid-sensing ion channels (ASICs) and Na+/H+ exchangers (NHE1) provide auxiliary proton disposal routes (19). Beyond its physicochemical effects, lactate also serves as the substrate for histone lysine lactylation, an epigenetic mark that couples glycolytic flux directly to gene expression (20); in HCC, lactylome analyses have linked lactylation to metabolic adaptation and PD-L1 stabilization, adding an epigenetic layer to acidity-driven immune evasion (21). Collectively, these mechanisms convert a metabolic byproduct into an active organizer of the immunosuppressive niche (**Figure 1**).

**Figure 1:**
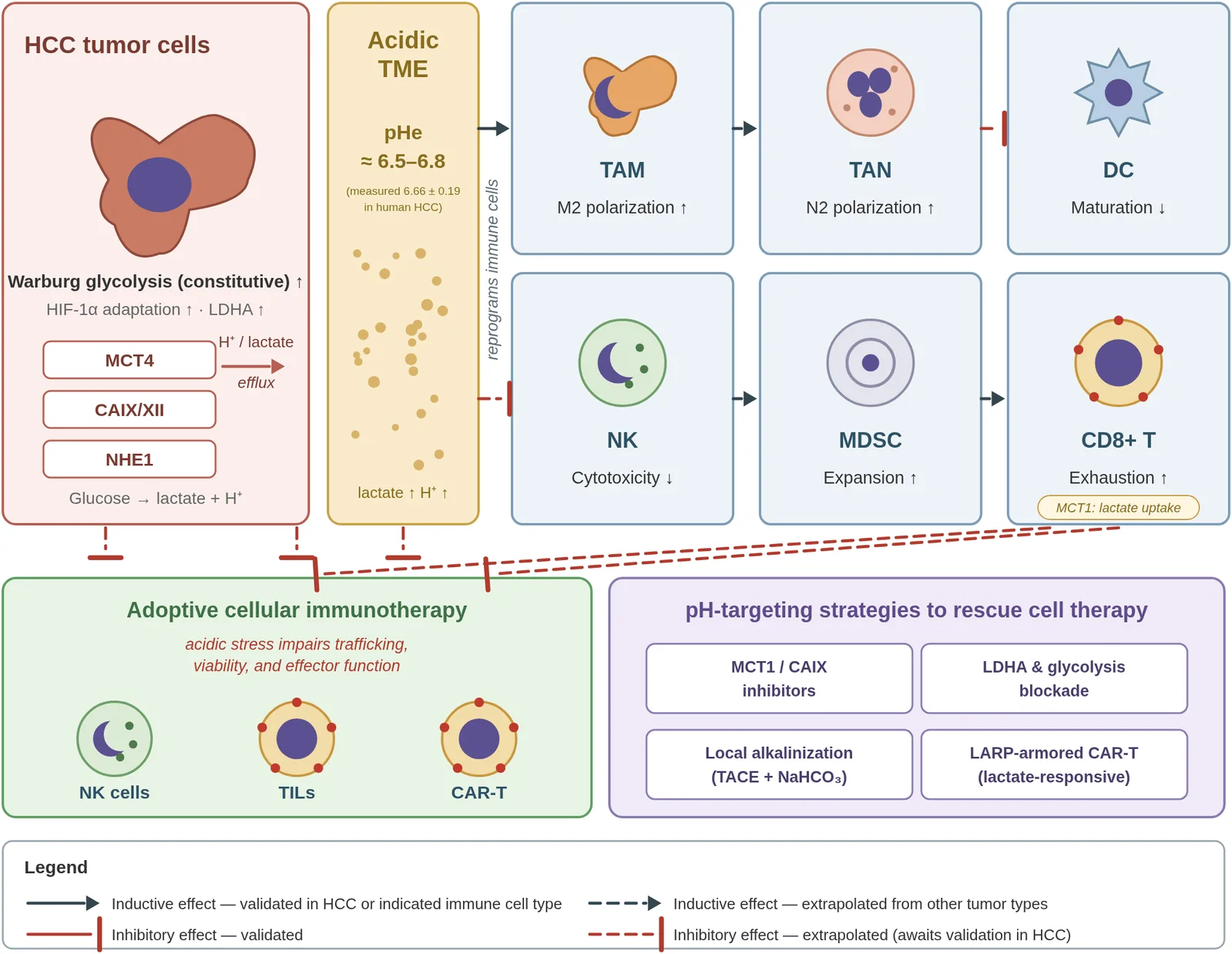
The acidic tumor microenvironment (TME) in hepatocellular carcinoma (HCC) reshapes the immune landscape and impedes adoptive cellular immunotherapy. HCC cells rely on constitutive aerobic glycolysis (the Warburg effect) and hypoxia-induced, HIF-1α-dependent glycolytic adaptation to produce lactate and protons, which are exported from HCC cells predominantly via the low-affinity monocarboxylate transporter MCT4 (whereas lactate uptake by infiltrating immune cells occurs mainly via the higher-affinity MCT1), together with carbonic anhydrases (CAIX/XII) and Na+/H+ exchanger 1 (NHE1), generating an extracellular acidic milieu (extracellular pH, pHe, approximately 6.5–6.8). The acidic niche reprograms tumor-infiltrating immune cells toward immunosuppressive or dysfunctional states: M2-polarized tumor-associated macrophages (TAMs; IL-10, TGF-β, VEGFA), N2-polarized tumor-associated neutrophils (TANs; CCL2, CCL17), poorly matured dendritic cells (DCs; reduced MHC-II and CD80/86), hypofunctional NK cells (reduced NKG2D and IFN-γ), expanded myeloid-derived suppressor cells (MDSCs; CCL26, ENTPD2), and exhausted CD8+ T cells (PD-1, TOX). It further impairs the trafficking, viability, and effector function of adoptively transferred NK cells, tumor-infiltrating lymphocytes (TILs), and CAR-T cells. pH-targeting strategies—including MCT1/CAIX inhibition, LDHA/glycolysis blockade, local alkalinization (e.g., TACE combined with sodium bicarbonate), and lactic acid-responsive promoter (LARP)-armored CAR-T cells—may restore the efficacy of cellular immunotherapy. Solid arrows indicate inductive effects and red T-bar lines (⊥) indicate inhibitory effects. Solid lines denote mechanisms experimentally validated in HCC or in the indicated immune cell type; dashed lines denote relationships extrapolated from other tumor types or experimental systems, whose validity in HCC remains to be established.

## 3 Cell-type-specific effects of the acidic niche

### 3.1 Tumor-associated macrophages

Immune cells sense extracellular acidification through ASICs and G protein-coupled receptors (GPR4, OGR1), transducing signals via cAMP, PKA, and MAPK cascades (22) that broadly inhibit effector T cell activation, suppress proinflammatory cytokine production, and promote peripheral tolerance (23, 24). TAMs are the most abundant and plastic immune population in this niche. Originating from circulating monocytes, they are reprogrammed by tumor-derived lactate and hypoxic stress toward an M2-like phenotype marked by CD163/CD206 upregulation and robust IL-10/TGF-β secretion, which directly impairs CD8+ T cell cytotoxicity and promotes Treg differentiation via FoxP3 upregulation, establishing a self-reinforcing suppressive circuit (25, 26). In human HCC, gut-microbiome-derived D-lactate enriched in the portal circulation drives M2 TAM polarization and remodels the immunosuppressive niche, providing HCC-specific evidence for lactate–macrophage crosstalk (27). Mechanistically, hypoxia-induced HMGB1 expression in HCC cells promotes tumor invasion by stimulating macrophage-derived IL-6 (28), while carbonic anhydrase XII—a key pH-regulatory enzyme—mediates the survival and pro-metastatic functions of macrophages in human HCC (29). The pro-tumorigenic functions of acid-reprogrammed TAMs extend to angiogenesis: in orthotopic models, acidified conditions drive TAM secretion of VEGFA and angiopoietin, accelerating vascularization, whereas blockade of M2 polarization attenuates angiogenesis and tumor growth. Importantly, as shown in non-HCC tumor models, acidic pH not only initiates but longitudinally maintains the M2 state, preventing reversion to tumoricidal M1 macrophages (26); whether this maintenance mechanism operates identically in HCC remains to be established—a rationale for combining pH buffering or lactate-export inhibition with strategies that repolarize TAMs. Two caveats remain: M1/M2 labels oversimplify the continuum of TAM states in human HCC (30), and lactate-versus-proton contributions to M2 polarization are rarely dissociated experimentally.

### 3.2 Tumor-associated neutrophils and dendritic cells

TANs polarize between anti-tumorigenic N1 and pro-tumorigenic N2 states depending on local metabolic and cytokine cues, and the acidic, hypoxic HCC milieu drives the balance progressively toward N2 as tumors advance. This N1/N2 framework rests largely on murine data; neutrophil states in human HCC are less defined, and specific surface markers are lacking. In human HCC, intratumoral neutrophils recruit macrophages and regulatory T cells via CCL2 and CCL17, promoting tumor progression and sorafenib resistance (31); CXCR2-dependent chemokine gradients sustain neutrophil influx and pro-tumoral polarization (32), and reciprocal crosstalk with cancer stem-like cells further reinforces this tumor-promoting loop (33). Acidosis and lactate then act as metabolic drivers of the N2 transition, reprogramming TANs to secrete immunosuppressive mediators, impair CD8+ T cell cytotoxicity, and remodel the extracellular matrix. In HCC specifically, tumor-derived lactate induces PD-L1 expression on neutrophils, contributing to lenvatinib resistance (34). In parallel, the acidic niche compromises dendritic cells, the master antigen-presenting cells. Tumor-derived lactic acid directly suppresses DC activation and antigen expression, downregulating MHC-II and co-stimulatory molecules (CD80/CD86) and culminating in defective T cell priming and peripheral anergy (35). These DC findings were obtained in non-HCC systems and await direct validation in HCC. Tumor-secreted IL-10 and VEGF synergize with hypoxia to enrich immature or tolerogenic DCs—including plasmacytoid DCs, whose intratumoral accumulation predicts poor prognosis after HCC resection and is driven by HIF-1α/adenosine signaling (36–38). Pharmacological targeting of glycolytic flux or lactate production can partially rejuvenate DC metabolic fitness, offering a route to restore antigen presentation in this niche. Moreover, cDC1, cDC2, and plasmacytoid DCs differ in function and likely in acid sensitivity (39), and acidosis effects on MHC-I cross-presentation remain underexplored.

### 3.3 Natural killer cells and myeloid-derived suppressor cells

NK cells are metabolically crippled in the acidic niche: extracellular acidosis and lactate induce mitochondrial dysfunction and apoptosis in liver-resident NK cells, as demonstrated in colorectal liver metastasis models (40); NK cells from HCC patients exhibit defective energy metabolism and motility (41), and hypoxia-driven HIF-1α stabilization rewires NK metabolism toward compensatory glycolysis, exacerbating exhaustion and diminishing IFN-γ secretion (42). This blockade, compounded by adenosine, IL-10, and TGF-β in the acidic milieu, is pharmacologically reversible: buffering pHe or inhibiting lactate exporters restores NK metabolic fitness and enhances tumor clearance in preclinical models (43, 44). Whether acidosis also impairs antibody-dependent cellular cytotoxicity (ADCC), and whether lactate or low pH per se drives NKG2D downregulation, remain unresolved. Conversely, the same niche expands and stabilizes MDSCs—immature myeloid precursors arrested in a highly suppressive state—whose circulating and intratumoral frequencies correlate with HCC progression, tumor burden, and systemic immune dysfunction (45, 46). MDSCs deplete L-arginine and L-cysteine via arginase-1 and iNOS, generate reactive oxygen and nitrogen species, and inhibit DC antigen presentation. Hypoxia recruits MDSCs to HCC via the chemokine CCL26 (47), and HIF-1α-dependent ENTPD2/CD39L1 expression amplifies glycolytic flux and immunosuppressive enzyme programs, locking MDSCs in a sustained immunoevasive state (48). Most lactate-driven MDSC expansion data come from non-HCC models, and monocytic versus polymorphonuclear subsets may differ in acid sensitivity (49). The divergent impact of acidosis—simultaneously crippling NK cytotoxicity while empowering MDSC-mediated suppression—positions extracellular pH as a central regulator of innate immune balance in HCC.

### 3.4 T Lymphocytes

CD8+ cytotoxic T lymphocytes execute tumor cell lysis via perforin/granzyme pathways and effector cytokines (IFN-γ, TNF-α), while CD4+ helper T cells coordinate immunity and macrophage polarization. Antigen recognition triggers a rapid switch to aerobic glycolysis to meet the bioenergetic demands of clonal expansion, rendering T cells exquisitely sensitive to acidic constraint (23). Models replicating the low-pH milieu in non-HCC systems consistently show suppressed cytokine production, diminished cytolytic granule exocytosis, and reduced proliferation across both subsets (50). Mechanistically, tumor-derived lactate is imported by T cells predominantly via MCT1 (51) and oxidized by lactate dehydrogenase to pyruvate, consuming NAD+ and elevating the cytosolic NADH/NAD+ ratio; this redox shift inhibits GAPDH, restrains glycolytic flux, and thereby limits T cell proliferation, as demonstrated in murine T cells (52). This mechanism does not imply competitive inhibition of mitochondrial respiration: depending on the metabolic state, lactate-derived pyruvate can instead enter the TCA cycle and fuel oxidative metabolism (53, 54), so the net effect of lactate on respiration is context-dependent. The resulting metabolic stress engages TOX-driven epigenetic remodeling that fixes the exhausted transcriptional program, as demonstrated in chronic infection and melanoma models (55), and MCT4 blockade has been shown to restore T cell infiltration and immune attack in HCC models (56). Acidic stress additionally skews CD4+ differentiation away from the pro-inflammatory Th1 lineage, attenuating cytotoxic support and M1 macrophage activation (23). Critically, Tregs are metabolically privileged in this niche: Foxp3 rewires Treg metabolism to oxidize lactate, allowing these cells to survive and suppress in the low-glucose, high-lactate conditions where effector T cells fail (57). This asymmetry—effector dysfunction alongside regulatory resilience—is the core immunological problem that any acidity-targeted intervention must solve.

The redox consequences of lactate must be parsed by redox couple. Lactate/pyruvate interconversion is coupled to the cytosolic NADH/NAD+ pool, which governs metabolic flux, whereas antioxidant defense depends on the NADPH/NADP+ pool that maintains reduced glutathione and thioredoxin systems (58). The oxidative pentose phosphate pathway (PPP) is the principal source of NADPH, and immune cells are functionally dependent on it (59, 60). Importantly, reduced glycolytic flux cannot be equated with reduced PPP flux: under oxidative stress, glucose-6-phosphate is acutely rerouted into the PPP to boost NADPH production (61). In the lactate-rich HCC niche, chronic glycolytic constraint may nonetheless limit PPP substrate supply, compromising NADPH-dependent ROS clearance and favoring T cell exhaustion; on the tumor side, the non-oxidative PPP enzyme transketolase counteracts oxidative stress and drives HCC progression (62). How lactate and acidosis reshape the PPP–NADPH axis in HCC-infiltrating immune cells, particularly in exhausted CD8^+^ T cells, remains an open question.

The effects described above are summarized in **Table 1**.

**Table 1:** Immune cell dysfunction in the acidic microenvironment of hepatocellular carcinoma (HCC).

Immune cell	Effect of acidic TME	Mechanisms of immune suppression	Key pathways	Therapeutic implications
Tumor-Associated Macrophages (TAMs)	M2 polarization; sustained immunosuppressive phenotype	IL-10 and TGF-β secretion; induction of Tregs; promotion of angiogenesis and EMT	HIF-1α, HMGB1–IL-6 axis, CA XII, CD163, CD206	Inhibit TAM recruitment; reprogram toward M1 phenotype
Tumor-Associated Neutrophils (TANs)	Shift to N2 phenotype; tumor-supportive functions	Suppression of cytotoxic T cells; promotion of angiogenesis and extracellular matrix remodeling	HMGB1–TLR4, NF-κB, p38 MAPK, CXCR2, CXCL2, CXCL5	Inhibit TAN recruitment or polarization; block chemokine signaling
Dendritic Cells (DCs)	Impaired maturation and antigen presentation	Downregulation of MHC-II and co-stimulatory molecules; accumulation of tolerogenic DCs	Lactate, VEGF, IL-10, HIF-1α	Restore DC metabolic fitness; combine with ICI therapy
Natural Killer (NK) Cells	Reduced cytotoxicity and activation	Decreased NKG2D expression; metabolic exhaustion; adenosine-mediated inhibition	HIF-1α, adenosine signaling, MCT transporters	Buffer extracellular pH; restore NK metabolism; adoptive NK cell therapy
Myeloid-Derived Suppressor Cells (MDSCs)	Functional stabilization and expansion	Arginine depletion; ROS/RNS production; inhibition of T cell activation	HIF-1α, CCL26, ENTPD2/CD39L1, lactate	Block MDSC recruitment or suppressive function; target metabolic support
T Lymphocytes (CD4+/CD8+)	Exhaustion and functional suppression	Impaired cytokine secretion; metabolic reprogramming; PD-1 upregulation	MCT1 (immune-cell lactate uptake), MCT4 (tumor-cell lactate efflux), PD-1, HIF-1α, TOX, histone lactylation	Inhibit lactate transport; buffer pH; combine with CAR-T or TIL therapy

Mechanisms listed for each cell type are annotated in the main text by evidence level—validated in human HCC, obtained in HCC experimental models, or extrapolated from other tumor types. Several limitations merit attention: most mechanistic data derive from preclinical models or *in vitro* cultures at fixed pH, which poorly recapitulate the dynamic, heterogeneous pH gradients of human HCC; the table necessarily groups findings across diverse experimental systems, potentially obscuring context-dependent differences in acid sensitivity; and the therapeutic implications listed remain largely speculative, as clinical translation of pH-targeting strategies is still in its infancy. Cell-type-specific caveats—including the M1/M2 oversimplification for TAMs, murine-based N1/N2 definitions for TANs, DC-subset and cross-presentation gaps, unresolved ADCC and NKG2D mechanisms for NK cells, and M-MDSC/PMN-MDSC distinctions—are discussed in Sections 3.1–3.4.

## 4 Adoptive cellular immunotherapy under acidic stress

ACI leverages ex vivo expansion, functional augmentation, or genetic engineering of autologous or allogeneic immune cells to circumvent tumor immune evasion (5). Clinical experience supports this approach: adjuvant infusion of autologous cytokine-induced killer cells after curative resection improved recurrence-free and overall survival in a large randomized study (63). Yet the three major platforms differ in their intrinsic sensitivity to acidity, and recognizing these differences is essential for rational engineering: NK cells are exquisitely acid-sensitive at the level of mitochondrial fitness, TILs succumb primarily to lactate-driven exhaustion, and CAR-T cells face compounded barriers of impaired trafficking, persistence, and metabolic fitness within the acidic niche.

### 4.1 NK cell-based therapy

NK cells provide rapid, MHC-unrestricted cytotoxicity but are profoundly suppressed in HCC patients owing to acidosis-driven metabolic impairment and activating receptor downregulation. Ex vivo-expanded and activated NK cells efficiently kill autologous HCC cells and control tumor growth in preclinical models (64), and cytokine priming regimens such as IL-12/15/18 can restore the anti-HCC activity of dysfunctional NK cells (65). Because NK cells are among the most acid-sensitive effectors, combining such ex vivo preconditioning with *in vivo* buffering of the acidic niche is a rational strategy to sustain NKG2D expression, preserve mitochondrial fitness, and enhance tumor clearance after infusion.

### 4.2 Tumor-infiltrating lymphocyte therapy

TIL therapy capitalizes on endogenous, tumor-reactive T cell clones isolated from resected HCC specimens and expanded ex vivo with IL-2. Randomized evidence that adoptive transfer of ex vivo-activated autologous lymphocytes lowers postsurgical HCC recurrence dates back to a landmark Lancet trial (66), and current TIL protocols aim to reproduce such effects with more defined, metabolically optimized products (67). Nevertheless, reinfused TILs face rapid metabolic competition and lactate-driven signaling that induce exhaustion and limit long-term persistence within the acidic TME. Ex vivo metabolic engineering (e.g., PPARγ modulation and glycolytic checkpoint tuning) and co-administration of TME-normalizing agents are being explored to improve resilience and sustain IFN-γ production under acidic stress.

### 4.3 Chimeric antigen receptor T cell therapy

CAR-T therapy enables MHC-independent recognition of tumor-associated antigens. GPC3-targeted CAR-T cells eliminate HCC cells in proportion to antigen surface density (68), and phase I trials have confirmed acceptable safety with early efficacy signals, including tumor regression, in advanced HCC (69); AFP-targeted constructs recognizing AFP-derived peptides in an HLA-restricted manner effectively inhibit tumor progression in preclinical models (70). Despite these advances, CAR-T persistence, trafficking, and metabolic fitness remain severely hindered by the acidic TME. Next-generation designs therefore incorporate armored features—pH-neutralizing enzyme secretion, metabolic checkpoint modulators, or chemokine receptors—to improve intratumoral trafficking. A conceptually important advance is the lactic acid-responsive promoter (LARP), which converts tumor acidity from an inhibitory barrier into an activation switch: CAR expression remains silent in normal tissues and is induced only within the acidic TME, combining potency with markedly reduced off-tumor toxicity (71). Such acidity-responsive designs exemplify a paradigm shift from resisting the acidic niche to exploiting it as a tumor-selective cue.

## 5 Critical perspectives and unresolved questions

### 5.1 Acidity versus lactate: two effectors that are rarely dissociated

Most studies treat the acidic TME as a single entity, yet extracellular acidosis and lactate exert mechanistically distinct effects. Protons act directly on ion channels and pH-sensing receptors, whereas lactate functions as a signaling molecule (via GPR81/GPR132), a metabolic substrate, and a lactylation donor (72). The distinction is therapeutically consequential: pH buffering alone cannot block lactate signaling or lactylation, and lactate at low concentrations can even fuel oxidative metabolism in effector T cells, with suppression emerging only at higher, tumor-relevant concentrations (73, 74). Future HCC studies should report pHe and lactate independently—ideally with *in vivo* measurement—rather than inferring one from the other, and intervention trials should specify which effector they target.

### 5.2 The metabolic double-edged sword of systemic blockade

Systemic glycolytic or lactate-transport blockade faces a fundamental selectivity problem: activated effector lymphocytes are themselves glycolysis-dependent, so LDHA or MCT inhibition can impair the very cells that immunotherapy seeks to unleash. The so-called ‘lactate paradox’—whereby lactate can fuel or suppress T cells depending on concentration and context—exemplifies this selectivity problem (73). This reasoning favors cell-intrinsic solutions: engineering infused cells for acidic resilience ex vivo, where metabolic interventions can be applied transiently and specifically, avoids the systemic toxicity and compensatory metabolic escape that have limited small-molecule approaches (7).

### 5.3 Local rather than systemic pH intervention

The strongest clinical evidence that acidity is therapeutically actionable in HCC comes from a local intervention: targeting intratumoral lactic acidosis with bicarbonate infusion combined with transarterial chemoembolization (TACE) improved local control of large HCC in cohort and randomized studies (75). This result supports two design principles for combining pH modulation with cell therapy. First, locoregional delivery (hepatic arterial infusion, nanoparticle formulations) can achieve tumor-restricted alkalinization without systemic disturbance. Second, sequencing matters: transient TME normalization before or during cell infusion may create a therapeutic window for engraftment, whereas chronic systemic buffering is neither necessary nor desirable. Prospective trials of TACE or hepatic arterial alkalinization combined with NK or CAR-T infusion are a logical, and currently unmet, next step.

### 5.4 Measurement and stratification gaps

Finally, the field lacks standardized tools to quantify the acidic niche in patients. Human HCC pHe and lactate are rarely measured directly; most evidence derives from murine models whose metabolic parameters differ from human tumors. Encouragingly, CEST MRI has enabled pHe mapping in preclinical liver cancer models (76) and, in a first patient study, has quantified intratumoral pHe in human HCC (16). Candidate stratifiers—serum LDH, lactate levels, glycolytic enzyme expression, and metabolic imaging such as hyperpolarized 13C-MRI—remain investigational (8). Whether acidity is a driver or merely a correlate of aggressive disease also remains unresolved, and the causal contribution of lactylation to immune dysfunction in human HCC requires functional validation beyond descriptive lactylome mapping (21). Closing these gaps is a prerequisite for biomarker-guided, acidity-targeted combination trials.

### 5.5 Making tumor acidity druggable: pharmacological and engineering strategies

The unresolved issues outlined above converge on one practical question: which nodes of the acidity axis can actually be targeted, and with what tools? We propose five druggable nodes, summarized in **Figure 2**, with their clinical-stage products compiled in **Table 2**. First, lactate production can be attenuated at its source: pyruvate dehydrogenase kinase inhibition with dichloroacetate diverts pyruvate toward mitochondrial oxidation and has completed early-phase testing with manageable toxicity (79). Second, lactate and proton export can be blocked at the monocarboxylate transporters: the first-in-class MCT1 inhibitor AZD3965 has completed a Phase I dose-escalation trial with an established recommended Phase 2 dose, although its lack of MCT4 activity mandates selection of MCT1-high, MCT4-low tumors (77). Third, the extracellular acidification machinery itself can be inhibited: the carbonic anhydrase IX inhibitor SLC-0111 has completed Phase I testing in advanced solid tumors and entered a Phase Ib trial in CAIX-positive pancreatic cancer (78). Fourth, proton-sensing receptors on immune cells (GPR65/TDAG8, GPR132, and related family members) remain largely untapped drug targets for directly reprogramming acidosis-sensing immune subsets (22). Fifth, and most relevant to cellular immunotherapy, acidity can be exploited as a tumor-selective filter rather than neutralized.

**Figure 2:**
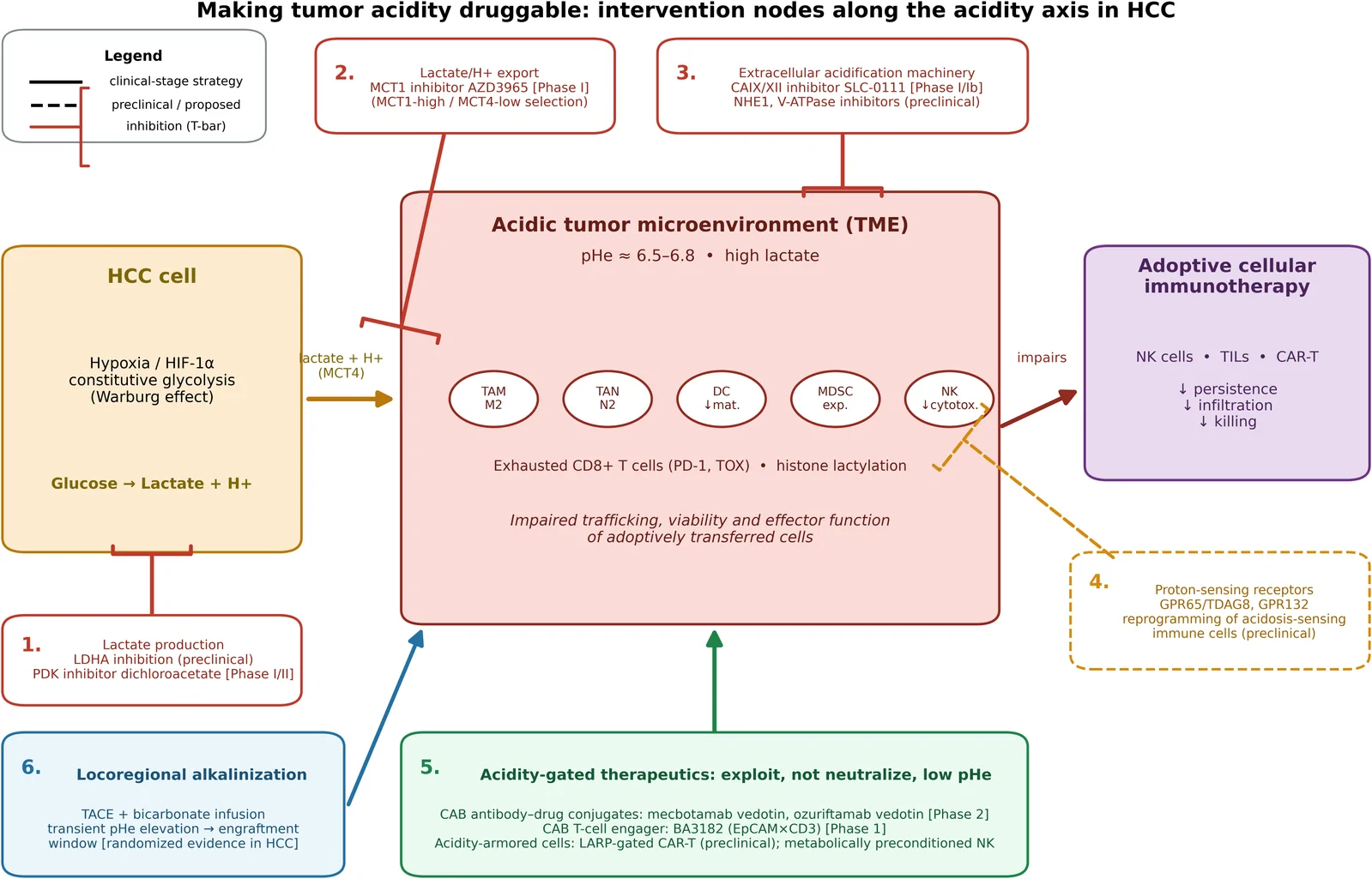
Making tumor acidity druggable: intervention nodes along the acidity axis in hepatocellular carcinoma (HCC). Lactate and protons produced by glycolytic HCC cells acidify the tumor microenvironment (extracellular pH, pHe, approximately 6.5–6.8) through five druggable nodes: (1) lactate production, inhibitable at LDHA or by redirecting pyruvate into mitochondria with pyruvate dehydrogenase kinase inhibitors (dichloroacetate); (2) lactate and proton export, blocked by monocarboxylate transporter inhibitors (MCT1: AZD3965); (3) the extracellular acidification machinery, inhibited at carbonic anhydrases IX/XII (SLC-0111), Na+/H+ exchanger 1 (NHE1), or vacuolar ATPase (V-ATPase); (4) proton-sensing receptors on immune cells (GPR65/TDAG8, GPR132) that translate acidosis into immune dysfunction; and (5) acidity-gated therapeutics that exploit, rather than neutralize, low pHe: conditionally active biologic (CAB) antibodies (the antibody-drug conjugates mecbotamab vedotin and ozuriftamab vedotin; the EpCAM x CD3 T-cell engager BA3182) and acidity-armored cellular products [lactic acid-responsive promoter (LARP)-gated CAR-T cells; metabolically preconditioned NK cells]. Locoregional alkalinization (transarterial chemoembolization combined with bicarbonate infusion) transiently raises pHe to create an engraftment window for adoptive cell transfer. Solid lines denote strategies with clinical-stage validation (**Table 2**); dashed lines denote preclinical or proposed strategies. Red T-bar lines (⊥) indicate inhibition.

**Table 2:** pH-targeting and acidity-armored products in clinical development or clinical proof-of-concept.

Product (class)	Modality	Mechanism of pH-sensing/acid-targeting	Cancer type(s)	Development stage (trial identifier)	Ref.
AZD3965	Small molecule (MCT1 inhibitor)	Blocks MCT1-mediated lactate/H+ transport; induces intracellular acidification of MCT1-high tumor cells	Advanced solid tumors and lymphoma (MCT1-high, MCT4-low selected)	Phase I completed (NCT01791595); recommended Phase 2 dose established	(77)
SLC-0111	Small molecule (CAIX/XII inhibitor)	Inhibits tumor-associated carbonic anhydrases; blocks extracellular acidification	Advanced solid tumors; CAIX-positive pancreatic cancer	Phase I completed (NCT02215850); Phase Ib ongoing (NCT03450018)	(78)
Dichloroacetate (DCA)	Small molecule (PDK inhibitor)	Diverts pyruvate from lactate production to mitochondrial oxidation; reduces tumor lactate output	Recurrent malignant brain tumors; locally advanced head and neck cancer (with chemoradiation)	Phase I/II completed (e.g., NCT01386632)	(79)
Sodium bicarbonate (TILA-TACE)	Buffer therapy (locoregional alkalinization)	Neutralizes intratumoral lactic acidosis via arterial bicarbonate infusion combined with TACE	Large hepatocellular carcinoma	Randomized controlled clinical study completed	(75)
Mecbotamab vedotin (BA3011)	Conditionally active biologic (CAB) ADC, anti-AXL	pH-gated target binding: active at acidic TME pHe (~6.0–6.7), minimal binding at physiological pH 7.4	AXL-positive sarcoma; NSCLC	Phase I/II and Phase II ongoing (NCT03425279; NCT04681131)	(80)
Ozuriftamab vedotin (BA3021)	CAB ADC, anti-ROR2	pH-gated target binding (as above)	ROR2-positive SCCHN, NSCLC, melanoma, TNBC	Phase I/II (NCT03504488); Phase II (NCT05271604); FDA Fast Track (SCCHN)	(81)
BA3182	Dual-conditionally active bispecific T-cell engager (CAB-EpCAM x CAB-CD3)	Both binding arms gated by acidic TME; >100-fold widened therapeutic index in preclinical models	Advanced adenocarcinomas (including cholangiocarcinoma)	Phase I ongoing (NCT05808634); early antitumor activity reported	(82)
CAIX CAR-T (G250-based)	CAR-T (first generation)	Targets carbonic anhydrase IX, the tumor pH-regulating enzyme	Metastatic renal cell carcinoma	Phase I/II completed; on-target, off-tumor hepatotoxicity managed by CAIX mAb pretreatment	(83)
LARP-armored CAR-T	Acidity-armored CAR-T	Lactic acid-responsive promoter restricts CAR expression to the acidic TME	Solid tumors (preclinical models)	Preclinical/IND-enabling; no acute hepatotoxicity in preclinical models	(71)

ADC, antibody-drug conjugate; CAIX/XII, carbonic anhydrase IX/XII; CAB, conditionally active biologic; CAR, chimeric antigen receptor; IND, investigational new drug; LARP, lactic acid-responsive promoter; MCT, monocarboxylate transporter; NSCLC, non-small cell lung cancer; PDK, pyruvate dehydrogenase kinase; SCCHN, squamous cell carcinoma of the head and neck; TACE, transarterial chemoembolization; TILA-TACE, targeting intratumoral lactic acidosis-TACE; TME, tumor microenvironment; TNBC, triple-negative breast cancer. Trial identifiers and status per ClinicalTrials.gov (accessed September 2026). None of the listed agents has yet been evaluated in an HCC-specific cohort except TILA-TACE; inclusion of a product does not imply proven efficacy in HCC but defines the translational toolkit discussed in Section 5.5.

This fifth strategy is now clinically real (**Table 2**). Conditionally active biologic (CAB) antibodies engineered to bind only at acidic extracellular pH (inflection near pH 6.6) have yielded two antibody-drug conjugates in Phase 2 trials, mecbotamab vedotin (anti-AXL) and ozuriftamab vedotin (anti-ROR2, FDA Fast Track in head and neck cancer), and a dual-conditionally-active EpCAM x CD3 T-cell engager (BA3182) in Phase I testing with early signals of antitumor activity and low rates of cytokine release syndrome (80–82). These agents demonstrate that acidity gating can widen the therapeutic index of modalities that otherwise fail through on-target, off-tumor toxicity. The same logic is reaching cell therapy from two directions. The first-generation CAIX-directed CAR-T experience in renal cell carcinoma, in which hepatotoxicity arose from CAIX expression on bile duct epithelium, stands as a direct cautionary precedent for targeting pH-regulating enzymes in the liver (83). Conversely, the lactic acid-responsive promoter (LARP) design restricts CAR expression to the acidic TME and eliminated acute hepatotoxicity in preclinical models, converting acidity from barrier to activation switch (71). In parallel, locoregional alkalinization with bicarbonate infusion combined with transarterial chemoembolization (TACE) remains the only pH-targeting intervention with randomized clinical evidence in HCC itself (75).

Mechanistically, these tools finally permit the acidity-versus-lactate dissociation that the field has lacked (Section 5.1). Proton-selective interventions (buffering, CAIX/NHE1/V-ATPase blockade, or agonism of proton-sensing receptors) can be contrasted with lactate-selective interventions (LDHA or MCT inhibition, and emerging agents targeting the writer and eraser machinery of lactylation), alone and in combination, to assign causality to each effector. For HCC, we propose a concrete translational sequence: first, stratify patients by intratumoral pHe using CEST MRI, already feasible in human liver tumors (16, 76); second, create a transient engraftment window by locoregional alkalinization during TACE (75); third, deliver acidity-armored effector cells, such as LARP-gated CAR-T or metabolically preconditioned NK cells, that are activated rather than incapacitated by the residual acidic niche (71). Anchoring each step to a measurable pharmacodynamic readout (tumor pHe, lactate concentration, and immune-cell lactylation) would convert the current descriptive literature into a mechanistically interpretable combination trial.

Two caveats temper this roadmap. No acidity-gated product has yet been tested in an HCC cohort, and the selectivity of pH-gated binding depends on the magnitude and spatial heterogeneity of tumor acidification, which is only beginning to be mapped in human HCC (16). Moreover, even pH-gated agents cannot fully exclude activity in non-malignant acidic niches such as inflamed or ischemic tissue. These caveats argue for HCC-specific validation, including pharmacodynamic pHe mapping, bile-duct and normal-liver safety monitoring, and acidity-stratified trial design, rather than against the strategy itself. Making acidity druggable is therefore less a search for new targets than a matching of existing, clinically tested tools to the acidic biology of HCC (**Figure 2**; **Table 2**).

## 6 Conclusion

The acidic TME of HCC is not a passive metabolic byproduct but an active, druggable determinant of immune dysfunction that disproportionately constrains adoptive cellular immunotherapy. Mechanistic understanding of how acidosis and lactate reprogram each immune subset is now substantial; the next advance must come from translation. We argue that the most promising direction is a dual strategy—ex vivo engineering of effector cells for acidic resilience, exemplified by metabolic preconditioning and acidity-responsive promoters, combined with locoregional, transient TME normalization to create a window for engraftment. Framing acidity as a patient-stratification biomarker and a therapeutic target, rather than background context, will be essential to convert the biological promise of cellular immunotherapy into durable clinical responses in HCC.
